# S100A12 drives inflammatory and metabolic reprogramming in sepsis-associated acute kidney injury

**DOI:** 10.3389/fmolb.2026.1741799

**Published:** 2026-01-23

**Authors:** Huanqin Liu, Yanan Lv, Qingjie Xue, Jikui Shi

**Affiliations:** 1 Department of Critical Care Medicine, Jining No.1 People’s Hospital, Jining, Shandong, China; 2 School of Clinical Medicine, Jining Medical University, Jining, Shandong, China; 3 School of Basic Medicine, Jining Medical University, Jining, Shandong, China

**Keywords:** biomarker, inflammation, metabolic reprogramming, RAGE, S100A12, sepsis, sepsis-associated acute kidney injury, TLR4

## Abstract

Sepsis-associated acute kidney injury (SA-AKI) is a severe complication of sepsis characterized by dysregulated inflammation, endothelial injury, and metabolic reprogramming. Among the numerous inflammatory mediators involved, S100 calcium-binding protein A12 (S100A12), a neutrophil-derived alarmin, has emerged as a key amplifier of receptor for advanced glycation end-products (RAGE) and toll-like receptor 4 (TLR4) signaling in this context. Through activation of these pathways, S100A12 drives inflammatory amplification, promotes cytokine release, pyroptotic and apoptotic cell death, endothelial dysfunction, and impaired tubular repair, thereby exacerbating renal injury. Experimental studies demonstrate that inhibition of S100A12 or blockade of its downstream signaling attenuates inflammation and tissue damage, whereas clinical evidence associates elevated circulating and urinary S100A12 levels with disease severity and adverse prognosis in sepsis. Collectively, current evidence positions S100A12 as both a mechanistic driver of inflammatory and metabolic reprogramming and a clinically actionable biomarker in SA-AKI. This review summarizes recent advances in the molecular biology and immunometabolic roles of S100A12 in SA-AKI, emphasizes its systemic versus kidney-specific effects, and discusses its translational potential as a biomarker and therapeutic target, highlighting opportunities and challenges for precision diagnostics and targeted therapies in sepsis-related organ injury.

## 1 Introduction

Sepsis-associated acute kidney injury (SA-AKI) is one of the most severe complications of sepsis, affecting up to half of critically ill patients and contributing substantially to morbidity and mortality (Gomez et al., 2014; Wang et al., 2021; Zarbock et al., 2023a). Despite advances in supportive care, the mortality of SA-AKI remains unacceptably high, often exceeding 50% (Kuwabara et al., 2022). Unlike other etiologies of acute kidney injury, SA-AKI arises from a complex interplay among dysregulated inflammation, microvascular dysfunction, and cellular metabolic reprogramming rather than from isolated ischemic or nephrotoxic insults (Cao et al., 2019; Hato and Dagher, 2025). These features complicate early diagnosis and limit the performance of conventional renal biomarkers, delaying timely therapeutic intervention (Zhu et al., 2021; Zarbock et al., 2023a; Hariri and Legrand, 2025).

S100 calcium-binding protein A12 (S100A12), a neutrophil-derived alarmin, has recently emerged as a key mediator and biomarker of inflammatory disorders. Through activation of the receptor for advanced glycation end-products (RAGE) and toll-like receptor 4 (TLR4), S100A12 amplifies innate immune signaling, promotes pyroptosis and apoptosis, impairs endothelial and tubular repair, and thereby exacerbates renal injury (Vallés et al., 2023; Zhou et al., 2023; Cross et al., 2024; Lin S. et al., 2025; Wang et al., 2025). Accumulating experimental and clinical evidence demonstrates that S100A12 levels are markedly elevated in sepsis, correlate with the severity of renal dysfunction, and may enable earlier detection of SA-AKI compared with conventional markers such as serum creatinine (He et al., 2024; Ostermann et al., 2024; Dubois, 2019). Moreover, preclinical studies indicate that inhibition of S100A12 signaling attenuates renal damage, highlighting its potential as a therapeutic target (Liliensiek et al., 2004; Kim C. H. et al., 2023; Vallés et al., 2023; Xia et al., 2024).

Given its dual role as both a mechanistic driver and a measurable biomarker, S100A12 represents a promising molecular link between innate immune activation and kidney injury in sepsis (Lin S. et al., 2025; Wu F. et al., 2025). This review summarizes current knowledge of S100A12 biology, its mechanistic contributions to the pathogenesis of SA-AKI, and its translational potential as a diagnostic and therapeutic target. In addition, it outlines key challenges and future directions required to translate these findings into clinical practice, including biomarker validation, mechanistic studies linking S100A12 to pyroptosis and immune signaling, and preclinical-to-clinical therapeutic development (e.g., small-molecule modulators or pathway blockade) (Lake et al., 2023; Ostermann et al., 2024; Yang et al., 2024; 2025). Notably, S100A12 is primate-specific; murine models therefore rely on transgenic or humanized S100A12 expression systems, which should be considered when interpreting experimental data.

## 2 Biology of S100A12

### 2.1 Structure and expression

Structurally, S100A12 is a small EF-hand calcium-binding protein that exerts multiple context-dependent functions. Also referred to as calgranulin C, it belongs to the S100 family of calcium-binding proteins and contains canonical EF-hand motifs (Moroz et al., 2001; Pietzsch and Hoppmann, 2009). This protein, with a molecular mass of approximately 10.4 kDa, comprises two EF-hand domains separated by a hinge region and a flexible, hydrophobic C-terminal tail (Moroz et al., 2003; Carvalho et al., 2020). The EF-hand domains coordinate divalent cations, most notably calcium (Ca^2+^) and zinc (Zn^2+^), which induce conformational rearrangements facilitating receptor interaction and oligomerization (Wang et al., 2019; Carvalho et al., 2020). Calcium binding promotes dimerization or higher-order oligomerization (e.g., tetramers, hexamers), essential for high-affinity receptor engagement—particularly with RAGE (Moroz et al., 2003; Wang et al., 2019).

S100A12 expression is largely restricted to cells of the myeloid lineage, with neutrophils serving as the predominant source, while monocytes and selected epithelial and endothelial cells express lower but inducible levels under inflammatory conditions (Lira-Junior et al., 2020; Li et al., 2022; Cross et al., 2024). During acute and chronic inflammation, transcriptional upregulation and extracellular release of S100A12 are markedly increased, and circulating concentrations correlate with disease activity and severity across conditions such as sepsis, autoimmune disease, and cardiovascular disorders (Xia et al., 2023). Pro-inflammatory cytokines such as tumor necrosis factor-alpha (TNF-α) and interleukin-1-beta (IL-1β) enhance S100A12 expression via nuclear factor kappa-light-chain-enhancer of activated B cells (NF-κB)–dependent transcriptional activation through CCAAT/enhancer-binding protein-beta (C/EBPβ) and activator protein-1 (AP-1) motifs (Ott et al., 2007; Li et al., 2014).

S100A12 is secreted via non-classical pathways and acts extracellularly as a damage-associated molecular pattern (DAMP). Its primary receptor is RAGE, though TLR4 is also implicated (Zhou et al., 2023; Cicchinelli et al., 2024). RAGE engagement activates mitogen-activated protein kinase (MAPK) and NF-κB cascades, amplifying leukocyte recruitment, cytokine release, and endothelial activation (Zhang et al., 2024; Xia et al., 2023). In SA-AKI, the S100A12–RAGE axis promotes endothelial dysfunction, oxidative stress, and tubular apoptosis, linking neutrophil activation to maladaptive renal inflammation (Aguilar et al., 2024; Qin et al., 2024).

Emerging evidence highlights the regulatory role of post-translational modifications (PTMs) and metal-ion interactions in fine-tuning S100A12 function. Zn^2+^ binding enhances oligomer stability and receptor affinity (Moroz et al., 2009), whereas oxidative modifications alter its extracellular activity under septic and inflammatory conditions (Foell et al., 2007; Goyette and Geczy, 2011). Collectively, these findings suggest that S100A12 acts as a dynamic molecular switch integrating neutrophil activation, oxidative stress, and microenvironmental cues, rather than a static pro-inflammatory mediator (Cicchinelli et al., 2024). Collectively, these structural features endow S100A12 with remarkable metal-ion-dependent conformational flexibility that underlies its biological activity. Notably, S100A12 is primate-specific; murine investigations therefore rely on transgenic or humanized models.

**FIGURE 1:**
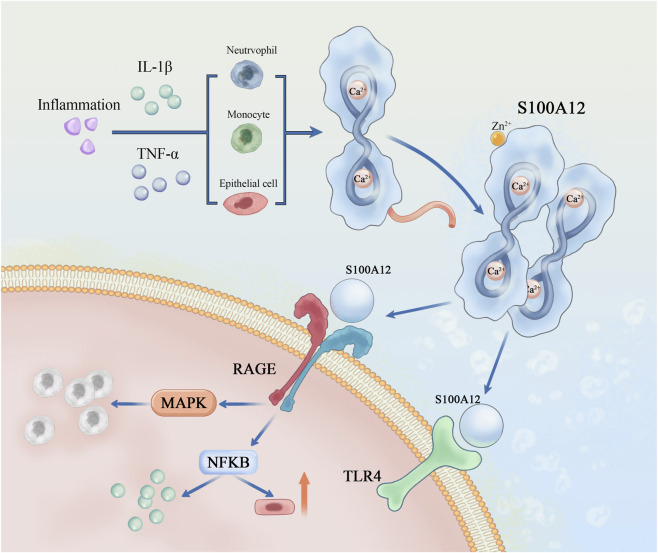
Structural representation of S100A12 and its metal ion–dependent oligomerization. This figure depicts the three-dimensional structure of S100A12, a calcium-binding protein containing two EF-hand motifs. Binding of Ca^2+^ induces conformational changes that promote dimer formation, whereas Zn^2+^ facilitates hexamer assembly and stabilizes higher-order structures. These metal ion–driven transitions enhance S100A12’s affinity for its receptors RAGE and TLR4 on cell membranes, providing the structural basis for its role in amplifying inflammatory responses in sepsis-associated acute kidney injury. Abbreviations: S100A12, S100 calcium-binding protein A12; RAGE, receptor for advanced glycation end-products; TLR4, toll-like receptor 4; MAPK, mitogen-activated protein kinase; NF-κB, nuclear factor kappa-light-chain-enhancer of activated B cells; Ca^2+^, calcium ion; Zn^2+^, zinc ion; EF-hand, helix–loop–helix calcium-binding motif.

To better illustrate these molecular features, Figure 1 summarizes the overall domain organization of S100A12 and highlights how Ca^2+^ and Zn^2+^ cooperatively stabilize its oligomeric states and enable receptor engagement.

### 2.2 Mechanisms in inflammation and immunity

S100A12 orchestrates multiple aspects of the inflammatory cascade in SA-AKI through interconnected mechanisms.

#### 2.2.1 NF-κB activation via RAGE

Binding of S100A12 to RAGE on tubular epithelial and endothelial cells activates MAPK and NF-κB pathways, inducing the transcription of pro-inflammatory cytokines such as IL-1β, interleukin-6 (IL-6), and TNF-α. This axis sustains inflammatory amplification and paracrine injury within the renal microenvironment (Tanaka et al., 2019; Curran and Kopp, 2022; Dong et al., 2022; Kang et al., 2025).

#### 2.2.2 TLR4/MyD88 engagement

Independent of RAGE, S100A12 can act as an endogenous ligand for TLR4. Engagement of TLR4 triggers myeloid differentiation primary response 88 (MyD88)-dependent signaling, resulting in NF-κB translocation and enhanced cytokine production, thereby amplifying monocyte activation and systemic inflammation (Foell et al., 2013; Jung et al., 2020; Kim H.-J. et al., 2023, p. 4).

#### 2.2.3 Leukocyte recruitment

S100A12 possesses chemoattractant activity, promoting the recruitment and activation of neutrophils and monocytes. This leads to local immune cell accumulation, tubular epithelial cytotoxicity, and perpetuation of inflammatory damage (Yang et al., 2001; Rouleau et al., 2003; Yan et al., 2008; Meijer et al., 2012).

#### 2.2.4 Pyroptosis via NLRP3 inflammasome

SA-AKI is characterized by robust NOD-, LRR- and pyrin domain-containing protein 3 (NLRP3) inflammasome activation, leading to caspase-1 activation, gasdermin D cleavage, and pyroptotic cell death. S100A12 signaling primes NF-κB for enhanced NLRP3 assembly, promoting IL-1β and interleukin-18 (IL-18) release and exacerbating renal inflammation. Although direct *in vivo* and *in vitro* evidence for S100A12-induced NLRP3 assembly remains limited, multiple studies have demonstrated that other S100 family members, particularly heterodimer of S100A8 and S100A9 (S100A8/A9), can prime the NLRP3 inflammasome through TLR4/NF-κB or reactive oxygen species (ROS)-dependent pathways. Thus, it is reasonable to infer a comparable mechanism for S100A12, by analogy to other S100 proteins (e.g., S100A8/A9) (Liu et al., 2022; 2024; Zhang et al., 2023; Kodi et al., 2024; Wu W. et al., 2025).

#### 2.2.5 Apoptosis via caspase-3

S100A12 promotes tubular epithelial apoptosis via caspase-3 activation, compromising epithelial integrity and renal function in SA-AKI (Hofmann Bowman et al., 2011). Experimental evidence from *in vitro* and *in vivo* models indicates that S100A12 can upregulate Fas and trigger caspase-3–dependent cell death, while clinical data show elevated soluble extracellular newly identified RAGE-binding protein (EN-RAGE) levels in sepsis and SA-AKI patients correlating with renal dysfunctions (Daffu et al., 2013; Cross et al., 2024; Kang et al., 2025). Together, these *in vitro*/*in vivo* and clinical observations support a role for S100A12 in both inflammatory amplification and tubular epithelial injury, highlighting its potential as a mechanistically relevant biomarker and therapeutic target.

#### 2.2.6 Dysregulated autophagy

S100A12 may impair protective autophagy in tubular epithelial cells via NF-κB and inflammasome signaling, shifting stressed cells toward maladaptive death pathways such as pyroptosis and apoptosis (Zhao S. et al., 2023; Wu W. et al., 2025). This dysregulation of autophagy contributes to the loss of tubular integrity and exacerbates renal injury in SA-AKI (Zhang, 2024). Both experimental and clinical evidence support a role for S100A12 in modulating autophagic responses and promoting maladaptive cell death, highlighting its potential as a mechanistically relevant biomarker and therapeutic target (Chu et al., 2025). Collectively, these findings indicate that S100A12 contributes to the pathogenesis of SA-AKI through multiple interrelated inflammatory and immune mechanisms.

To provide a concise overview, the major signaling axes and downstream effects of S100A12 are summarized in Table1.

**TABLE 1:** Mechanistic roles of S100A12 in inflammation and immunity during sepsis-associated acute kidney injury (SA-AKI).

Mechanistic axis	Key pathway/Receptors	Molecular and cellular effects	Representative evidence	Pathophysiological outcome in SA-AKI
NF-κB activation via RAGE	S100A12–RAGE → MAPK/NF-κB	Upregulation of IL-1β, IL-6, TNF-α; sustained inflammatory amplification and paracrine injury within renal microenvironment	Foell et al. (2013), Curran and Kopp (2022), Dong et al. (2022), Kang et al. (2025)	Persistent cytokine storm, microvascular inflammation, and tubular damage
TLR4/MyD88 engagement	S100A12–TLR4 → MyD88-NF-κB axis	Activation of NF-κB, promotion of monocyte and macrophage inflammatory responses, amplification of systemic inflammation	(Yang et al., 2001; Jung et al., 2020; Kim H.-J. et al., 2023, p. 4)	Enhanced leukocyte activation and cytokine-driven renal inflammation
Leukocyte recruitment	S100A12 as chemoattractant	Chemotactic recruitment and activation of neutrophils/monocytes, induction of cytotoxicity toward tubular cells	(Rouleau et al., 2003; Yan et al., 2008; Meijer et al., 2012; Liu et al., 2022)	Immune cell infiltration, sustained tissue injury
Pyroptosis via NLRP3 inflammasome	S100A12 → NF-κB priming → NLRP3-caspase-1-GSDMD	Promotes IL-1β/IL-18 release, gasdermin D–mediated pore formation, and pyroptotic cell death; analogous to S100A8/A9 mechanisms	(Hofmann Bowman et al., 2011; Zhang et al., 2023; Kodi et al., 2024; Liu et al., 2024; Wu W. et al., 2025)	Exacerbated inflammatory cell death and renal tissue necrosis
Apoptosis via caspase-3	S100A12 → Fas-caspase-3 pathway	Induces tubular epithelial apoptosis, loss of epithelial barrier integrity; elevated serum EN-RAGE correlates with renal dysfunction	(Daffu et al., 2013; Foell et al., 2013; Zhao S. et al., 2023; Cross et al., 2024)	Tubular epithelial loss, renal dysfunction
Dysregulated autophagy	S100A12 → NF-κB/inflammasome axis	Impairs protective autophagy, shifts toward pyroptosis/apoptosis under stress	(Hofmann Bowman et al., 2011; Cunden et al., 2016; Zhang, 2024; Chu et al., 2025)	Maladaptive cell death and worsening tubular injury

Abbreviations: SA-AKI, sepsis-associated acute kidney injury; S100A12, S100 calcium-binding protein A12; RAGE, receptor for advanced glycation end-products; TLR4, Toll-like receptor 4; MyD88, myeloid differentiation primary response 88; NF-κB, nuclear factor kappa-light-chain-enhancer of activated B cells; MAPK, mitogen-activated protein kinase; IL, interleukin; TNF-α, tumor necrosis factor alpha; NLRP3, NOD-, LRR-, and pyrin domain-containing protein 3; GSDMD, gasdermin D; EN-RAGE, extracellular newly identified RAGE-binding protein; Fas, Fas receptor; ROS, reactive oxygen species; DAMPs, damage-associated molecular patterns.

### 2.3 Physiological and pathological roles

#### 2.3.1 Role in innate immunity

Under physiological conditions, S100A12 contributes to antimicrobial defense as part of the innate immune response. Its calcium- and zinc-dependent conformational changes enable direct interaction with microbial components and regulation of neutrophil adhesion, migration, and degranulation (Cunden et al., 2016). Extracellularly, S100A12 acts as a DAMP, engaging receptors such as RAGE and TLR4 to amplify immune signaling, enhance leukocyte recruitment, and stimulate cytokine secretion (Xia et al., 2023). These functions underscore its dual role as both an antimicrobial effector and an immunomodulatory signal in innate immunity (Singh and Ali, 2022).

#### 2.3.2 Contribution to systemic inflammation and tissue injury

While protective during localized infection, uncontrolled or sustained S100A12 release drives systemic inflammation. Elevated circulating levels of S100A12 are strongly associated with multi-organ dysfunction and poor outcomes in sepsis. Persistent activation of the S100A12-RAGE/TLR4 axis induces endothelial dysfunction, oxidative stress, and microvascular barrier disruption, thereby impairing tissue perfusion (Zhang, 2024). In the kidney, these processes promote tubular apoptosis, hinder reparative responses, and exacerbate inflammatory injury, contributing directly to SA-AKI pathogenesis (Arendshorst et al., 2024). Beyond the kidney, excessive S100A12 activity has been implicated in cardiovascular disease, autoimmune disorders, and other chronic inflammatory conditions, reflecting its broader role as a mediator of systemic tissue damage (Zhou et al., 2023).

## 3 Pathophysiological role of S100A12 in SA-AKI

**FIGURE 2:**
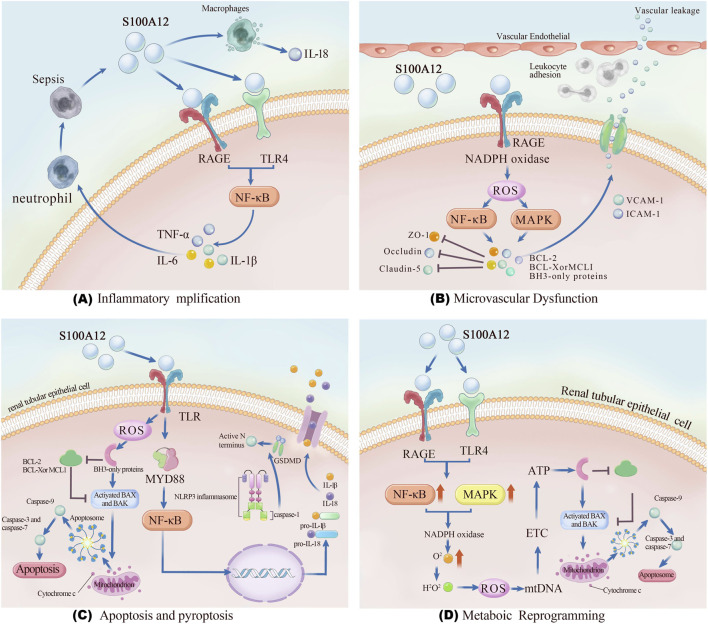
Schematic illustration of the multifaceted pathogenic mechanisms of S100A12 in sepsis-associated acute kidney injury. **(A)** Inflammatory amplification: S100A12 released from activated neutrophils and macrophages binds to RAGE and TLR4, activating NF-κB signaling and promoting the secretion of pro-inflammatory cytokines including IL-1β, IL-6, and TNF-α. **(B)** Microvascular dysfunction: S100A12 enhances endothelial activation via RAGE–NADPH oxidase–ROS signaling, upregulating VCAM-1 and ICAM-1 expression and disrupting tight junction proteins (ZO-1, occludin, claudin-5), leading to vascular leakage. **(C)** Apoptosis and pyroptosis: In renal tubular epithelial cells, S100A12 triggers TLR-mediated ROS accumulation and MYD88/NF-κB activation, promoting mitochondrial apoptosis and NLRP3 inflammasome-dependent pyroptosis. **(D)** Metabolic reprogramming: Through RAGE/TLR4–MAPK/NF-κB signaling, S100A12 induces mitochondrial oxidative stress, electron transport chain impairment, ATP depletion, and cytochrome c release, contributing to energy failure and tubular injury. Abbreviations: AKI, acute kidney injury; RAGE, receptor for advanced glycation end-products; TLR4, toll-like receptor 4; ROS, reactive oxygen species; VCAM-1, vascular cell adhesion molecule 1; ICAM-1, intercellular adhesion molecule 1; ZO-1, zonula occludens-1; NF-κB, nuclear factor kappa-light-chain-enhancer of activated B cells; MAPK, mitogen-activated protein kinase; NLRP3, NOD-like receptor family pyrin domain containing 3.

S100A12 participates in multiple pathogenic processes of SA-AKI, including inflammatory amplification, endothelial dysfunction, cell death, and metabolic reprogramming (Figure 2).

### 3.1 Inflammatory amplification

#### 3.1.1 Cytokine storm and neutrophil activation

SA-AKI is tightly linked to systemic hyperinflammation, in which S100A12 functions as a potent neutrophil-derived alarmin (Motomura et al., 2021). Upon release, S100A12 binds to the RAGE and TLR4, amplifying NF-κB–dependent cytokine production and driving a cytokine storm characterized by elevated IL-1β, IL-6, and TNF-α (Singh and Ali, 2022; Xia et al., 2023). This cascade perpetuates neutrophil activation, enhances degranulation, and aggravates renal injury (Motomura et al., 2021).

#### 3.1.2 Cross-talk with macrophages and endothelial cells

S100A12 also mediates cross-talk among neutrophils, macrophages, and endothelial cells. By activating macrophages, it promotes inflammasome priming and IL-18 release, further amplifying renal inflammation (Lenga Ma Bonda et al., 2022; Islamuddin and Qin, 2024). In parallel, S100A12 stimulates endothelial activation and upregulates adhesion molecules such as intercellular adhesion molecule-1 (ICAM-1) and vascular cell adhesion molecule-1 (VCAM-1), facilitating leukocyte adhesion and microvascular obstruction within the renal circulation (Kong et al., 2018; Haydinger et al., 2023, p. 1).

### 3.2 Microvascular dysfunction

#### 3.2.1 Endothelial barrier disruption

The S100A12–RAGE axis promotes endothelial dysfunction by increasing ROS-dependent oxidative stress and suppressing tight-junction proteins, culminating in barrier failure (Rojas et al., 2024). The resulting vascular leakage and microcirculatory collapse impair renal perfusion, independent of systemic hypotension (McMullan et al., 2024).

#### 3.2.2 Leukocyte adhesion and microcirculatory impairment

S100A12 enhances leukocyte rolling and adhesion within peritubular capillaries, aggravating microvascular congestion and impairing oxygen delivery (Yang et al., 2007). This imbalance between oxygen supply and metabolic demand contributes to the non-ischemic nature of SA-AKI (Sutton, 2009).

### 3.3 Cellular injury and death

#### 3.3.1 Pyroptosis and apoptosis in renal tubular cells

At the tubular level, S100A12 promotes both pyroptosis and apoptosis. NF-κB–driven activation of the NLRP3 inflammasome induces caspase-1 cleavage, gasdermin D pore formation, and pyroptotic cytokine release. Concurrently, S100A12 signaling triggers caspase-3–dependent apoptosis, further compromising tubular epithelial integrity.

#### 3.3.2 Impaired tubular repair and regeneration

Persistent S100A12 activation interferes with reparative signaling pathways (Xu et al., 2022). Excessive inflammation suppresses tubular proliferation and delays epithelial regeneration (Li et al., 2023), leading to incomplete recovery, progression to chronic kidney disease, and increased long-term morbidity (Lake et al., 2023).

### 3.4 Metabolic reprogramming

#### 3.4.1 Mitochondrial dysfunction

Mitochondrial injury is a hallmark of SA-AKI (Zhang et al., 2021). S100A12 exacerbates mitochondrial ROS generation and disrupts electron transport chain activity, resulting in reduced adenosine triphosphate (ATP) production (Su et al., 2023). This metabolic stress accelerates tubular apoptosis and undermines adaptive repair mechanisms (Rashid et al., 2023).

#### 3.4.2 Links between energy metabolism and kidney injury

Multi-omics studies reveal metabolic reprogramming as a defining feature of SA-AKI, characterized by suppressed fatty acid oxidation and disordered amino acid metabolism (Zhao L. et al., 2023). S100A12-driven inflammation may act synergistically with these metabolic derangements to aggravate energy deficits in tubular cells and heightening renal vulnerability to sepsis-induced injury (Wang et al., 2024).

### 3.5 Crosstalk between S100A12 and other DAMPs

Beyond its independent pro-inflammatory activity, S100A12 likely operates within a broader damage-associated molecular pattern (DAMP) network during sepsis and sepsis-associated AKI. Among these DAMPs, high-mobility group box 1 (HMGB1) is particularly relevant, as both S100A12 and HMGB1 are released during cellular stress and tissue injury and share key pattern-recognition receptors, including the RAGE and TLR4. Engagement of these shared receptors can converge on NF-κB and MAPK signaling pathways, leading to amplified cytokine production, endothelial activation, and sustained inflammatory responses (Rai et al., 2022; Cicchinelli et al., 2024).

Experimental studies in sepsis models suggest that DAMPs may act sequentially or synergistically, with early neutrophil-derived mediators such as S100A12 priming the inflammatory milieu, thereby enhancing cellular responsiveness to late mediators such as HMGB1. Although direct evidence for S100A12–HMGB1 cooperation in SA-AKI remains limited, this conceptual framework supports the notion that S100A12 functions as part of an integrated DAMP signaling network rather than as an isolated effector (Ludes et al., 2021; Huang et al., 2025; Lin H. et al., 2025).

### 3.6 Summary

Collectively, S100A12 acts as a central mediator linking systemic sepsis-induced inflammation to renal tissue injury (Ludes et al., 2021). By amplifying cytokine cascades, disrupting microvascular integrity, promoting tubular cell death, and exacerbating metabolic dysfunction, S100A12 contributes to both the onset and progression of SA-AKI (Kounatidis et al., 2024; Legrand et al., 2024; Zhang, 2024; Xia et al., 2023). These multifaceted effects underscore its dual role as a mechanistic driver of renal injury and a potential therapeutic and prognostic biomarker in sepsis-associated organ dysfunction.

## 4 Evidence from preclinical and clinical studies

### 4.1 Preclinical studies

Preclinical studies using transgenic mice that express human S100A12 (mice lack an endogenous S100A12 gene) demonstrate that, during sepsis, renal upregulation of human S100A12 is closely associated with tubular injury, neutrophil infiltration, and activation of pro-inflammatory pathways; genetic or antibody-mediated suppression of S100A12 attenuates apoptosis and improves renal function (Hofmann Bowman et al., 2010; Gawdzik et al., 2011; Bagheri, 2022). Complementary *in vitro* studies using human renal tubular epithelial cells and monocytes further corroborate these findings: small interfering RNA (siRNA)–mediated knockdown or antibody blockade of S100A12 significantly reduces lipopolysaccharide (LPS)-induced cytokine release and apoptosis (Foell et al., 2013; Zhang Z. et al., 2020).

### 4.2 Clinical observational studies

Clinical studies have confirmed these experimental findings, demonstrating that elevated circulating and urinary S100A12 levels are associated with both the presence and severity of SA-AKI (Dubois, 2019). In septic patient cohorts, plasma S100A12 concentrations were markedly higher in individuals who developed AKI and correlated with the degree of renal dysfunction (Wu et al., 2022). Notably, urinary S100A12 levels increased early during sepsis-preceding elevations in conventional renal biomarkers such as serum creatinine (Li et al., 2022). Furthermore, high S100A12 levels predicted worse outcomes, including increased mortality and the need for renal replacement therapy (Dubois, 2019), underscoring its potential as an early diagnostic and prognostic marker in septic patients.

### 4.3 Comparison with conventional biomarkers

Compared with established renal biomarkers such as serum creatinine, neutrophil gelatinase-associated lipocalin (NGAL), and kidney injury molecule-1 (KIM-1), S100A12 offers several key advantages. First, S100A12 rises early in the course of sepsis, allowing detection of renal injury before measurable changes in serum creatinine (Zhang C.-F. et al., 2020; Ortín-Bustillo et al., 2023). Second, its strong association with systemic inflammation and immune activation provides a mechanistic link between immune dysregulation and renal dysfunction, which conventional biomarkers lack (Wang et al., 2018; Seibert et al., 2021). Finally, studies indicate that S100A12 exhibits superior diagnostic performance, with higher sensitivity and specificity for predicting SA-AKI compared to NGAL and KIM-1 (Ortín-Bustillo et al., 2023; Brozat et al., 2024). These findings suggest that S100A12 may enable earlier clinical intervention and improved outcome stratification in patients with sepsis-related kidney injury.

### 4.4 Systemic versus kidney-specific effects of S100A12

An important unresolved issue is the distinction between systemic and organ-specific effects of S100A12 during sepsis (Raz, 2007). Circulating S100A12 primarily reflects systemic neutrophil activation and has been implicated in widespread endothelial dysfunction, microvascular injury, and cytokine amplification across multiple organs (Bertheloot and Latz, 2017; Cicchinelli et al., 2024). These systemic effects may indirectly contribute to renal hypoperfusion, inflammation, and susceptibility to acute kidney injury (Bertheloot and Latz, 2017; Lin H. et al., 2025).

In contrast, organ-specific effects within the kidney may arise from local interactions between S100A12 and renal tubular epithelial cells, glomerular or peritubular endothelial cells, and resident immune cells, potentially via RAGE- or TLR4-dependent signaling (Gustot, 2011; Lin H. et al., 2025). Such localized signaling could promote tubular inflammation, cell death, and metabolic dysfunction independently of systemic hemodynamic changes (Xie et al., 2013). However, current clinical and experimental studies often lack the spatial or temporal resolution required to clearly disentangle systemic from kidney-specific actions of S100A12 (Gustot, 2011; Cicchinelli et al., 2024). Future studies employing compartment-specific sampling, kidney-targeted experimental models, or spatial transcriptomic approaches may help clarify these distinct contributions (Cicchinelli et al., 2024).

## 5 Translational and therapeutic implications of S100A12 in SA-AKI

### 5.1 Diagnostic and prognostic value

#### 5.1.1 Risk stratification in septic patients

S100A12 has emerged as a promising biomarker for early detection and risk stratification in SA-AKI. Elevated admission plasma S100A12 correlates with increased mortality and multi-organ dysfunction in septic patients; for example, Dubois et al. reported that higher S100A12 levels at presentation identified septic shock patients at greater risk of death (Dubois, 2019). In addition, combining S100A12 with other clinically used markers (e.g., cardiac enzymes) improves the prediction of sepsis-related cardiac injury, supporting its broader prognostic utility in critical illness (Wu F. et al., 2025).

#### 5.1.2 Integration into biomarker panels

Integrating S100A12 into multi-analyte panels enhances early diagnosis and prognosis of SA-AKI. When used together with conventional renal biomarkers such as serum creatinine and NGAL, S100A12 increases sensitivity and specificity for detecting incipient renal injury, thereby enabling timelier intervention and risk-aligned management (Xie et al., 2021).

### 5.2 Therapeutic targeting of S100A12 pathways

#### 5.2.1 Neutralizing antibodies and small-molecule inhibitors

Therapeutic strategies that neutralize S100A12 or modulate its activity show promise in SA-AKI. Quinoline-3-carboxamide derivatives (e.g., ABR-215757) have been identified as small-molecule inhibitors capable of attenuating S100A12-driven inflammation and organ injury in preclinical settings, supporting targetability of this pathway (Yan et al., 2013).

#### 5.2.2 Modulation of RAGE/TLR4 signaling

S100A12 exerts pro-inflammatory effects primarily via the receptor for RAGE and TLR4. Pharmacologic interruption of these axes can blunt downstream signaling and tissue injury. FPS-ZM1, a high-affinity RAGE inhibitor, reduces inflammatory cytokine release and oxidative stress by blocking S100A12–RAGE interaction in experimental models (Shen et al., 2017). Likewise, interventions aimed at TLR4 signaling mitigate S100A12-initiated inflammatory cascades and have been proposed as a complementary strategy in SA-AKI (Vallés et al., 2023; Niu et al., 2024). Collectively, these findings support pharmacologic blockade of S100A12 and its downstream signaling as a rational and potentially translatable approach in SA-AKI. For comparison across modalities, major therapeutic strategies targeting the S100A12–RAGE/TLR4 axis are summarized in Table 2.

**TABLE 2:** Representative therapeutic strategies targeting S100A12 and the downstream RAGE/TLR4 axis.

Therapeutic strategy	Target/mechanism of action	Representative agents/interventions	Experimental/clinical evidence	Observed or proposed effect in SA-AKI
Neutralizing antibodies and small-molecule inhibitors	Direct inhibition of S100A12 activity; blockade of ligand–receptor interaction	Quinoline-3-carboxamide derivatives (e.g., **ABR-215757**); S100A12-neutralizing antibodies	Preclinical studies demonstrate suppression of S100A12-mediated inflammation and organ injury in models of sepsis and autoimmunity (Shen et al., 2017)	↓Pro-inflammatory cytokines (IL-1β, IL-6, TNF-α); ↓ oxidative stress; ↓ tissue injury
Modulation of RAGE signaling	Inhibition of S100A12-RAGE interaction; attenuation of downstream NF-κB/MAPK activation	**FPS-ZM1** (high-affinity RAGE inhibitor)	Experimental data show FPS-ZM1 reduces inflammatory cytokine release and oxidative stress by preventing S100A12-RAGE binding (Niu et al., 2024)	↓ Inflammatory amplification; ↓ microvascular dysfunction; protection of tubular epithelial cells
Modulation of TLR4 signaling	Suppression of S100A12-induced TLR4/MyD88-NF-κB axis	TLR4 antagonists (e.g., TAK-242, Eritoran)	Preclinical evidence supports inhibition of TLR4-dependent cytokine cascade and leukocyte activation (Ewaisha and Anderson, 2023; Vallés et al., 2023)	↓ Systemic inflammation; ↓ renal cytokine burden; attenuation of tubular injury

Abbreviations: SA-AKI, sepsis-associated acute kidney injury; S100A12, S100 calcium-binding protein A12; RAGE, receptor for advanced glycation end-products; TLR4, Toll-like receptor 4; NF-κB, nuclear factor kappa-light-chain-enhancer of activated B cells; MAPK, mitogen-activated protein kinase; IL, interleukin; TNF-α, tumor necrosis factor alpha; ROS, reactive oxygen species; DAMPs, damage-associated molecular patterns; ABR-215757, paquinimod; FPS-ZM1, RAGE, inhibitor; TAK-242, TLR4 inhibitor; Eritoran, TLR4 antagonist. Bold text highlights key inflammatory mediators or biological effects, and arrows indicate the direction of change.

#### 5.2.3 Challenges and safety considerations

Despite encouraging signals, several issues warrant careful evaluation before clinical translation. Specificity and off-target effects of neutralizing antibodies and small-molecule inhibitors must be rigorously profiled to minimize unintended interactions and efficacy loss; long-term safety and durability also require confirmation in well-designed trials. Methodological frameworks that apply orthogonal assays to characterize off-target liabilities can strengthen safety assessment paradigms (Wang et al., 2025). Moreover, redundancy and compensation within inflammatory networks mean that targeting S100A12 (and related ligands such as S100A8/A9) or their receptors (RAGE/TLR4) should balance efficacy with preservation of essential host defenses, a principle underscored by experience in autoimmune indications (Ewaisha and Anderson, 2023).

## 6 Current challenges and knowledge gaps

Despite compelling preclinical and early clinical evidence supporting the role of S100A12 in SA-AKI, several key challenges limit its translational applicability and clinical implementation.

### 6.1 Heterogeneity of clinical studies

Existing studies on S100A12 in SA-AKI are highly heterogeneous regarding patient selection, sepsis etiology, sampling time-points, and outcome definitions. Many cohorts include mixed intensive care unit (ICU) populations with varying organ dysfunction, obscuring the specific contribution of S100A12 to renal injury. Endpoints are inconsistently defined—from biochemical AKI criteria (serum creatinine/urine output) to composite organ dysfunction scores—further reducing comparability and hindering meta-analytic synthesis (Zarbock et al., 2023b; Legrand et al., 2024). To enable clinical validation, future studies should adopt standardized, patient-centered outcomes, prespecified sampling windows, and multicenter cohorts incorporating subgroup and sensitivity analyses to address confounding and heterogeneity (Ortín-Bustillo et al., 2023; Baeseman et al., 2024; Wu F. et al., 2025).

### 6.2 Lack of large-scale, prospective validation

Most available evidence is derived from small, single-center, or retrospective cohorts, which restricts the evaluation of S100A12’s predictive value in early-stage SA-AKI and risk stratification. Well-designed, prospective, multicenter studies are required to establish clinically meaningful thresholds, quantify inter-individual variability, and assess reproducibility across diverse populations differing in age, comorbidities, and sepsis etiologies (Legrand et al., 2024; Llitjos et al., 2024). Moreover, rigorous studies should clarify the incremental value of S100A12 over established kidney injury markers such as NGAL, KIM-1, and the cell-cycle arrest biomarker pair tissue inhibitor of metalloproteinases-2 × insulin-like growth factor-binding protein 7 ([TIMP-2]·[IGFBP7]) (Baeseman et al., 2024; Ostermann et al., 2024). Without harmonized protocols and prospective data, reliable integration of S100A12 into clinical workflows remains challenging. Future research should prioritize multicenter, preregistered validation cohorts and randomized trial sub-studies to confirm predictive capacity, define actionable thresholds, and clarify additive value beyond conventional biomarkers.

### 6.3 Limitations in translating animal data to humans

Translational studies face a major biological limitation: S100A12 is primate-specific, and mice lack an endogenous ortholog, complicating direct extrapolation from conventional murine models to human SA-AKI. Humanized or transgenic mice expressing human S100A12 provide valuable tools for mechanistic exploration but remain technically demanding and not widely available (Foell et al., 2007; Oesterle and Hofmann Bowman, 2015). Furthermore, interspecies differences in immune composition, renal metabolism, and inflammatory signaling restrict the translation of preclinical interventions—such as soluble receptor for advanced glycation end-products (sRAGE) or inflammasome inhibitors—to human settings (Russo et al., 2021; Zarbock et al., 2023b). Bridging this gap will require integrating humanized animal models, kidney organoids, and multi-omics platforms to more accurately recapitulate human disease biology.

### 6.4 Need for standardized assays for S100A12 detection

Although commercial enzyme-linked immunosorbent assay (ELISA) kits allow quantification of S100A12 in plasma, serum, and urine, significant analytical and preanalytical variability limits reproducibility. Factors such as blood collection technique, anticoagulant type, centrifugation parameters, storage conditions, and freeze–thaw cycles significantly affect measured concentrations and contribute to inter-laboratory inconsistency (O’Bryant et al., 2015; Agrawal et al., 2018). Assays from different manufacturers also vary in calibration standards, antibody specificity, and sensitivity, and published data on intra- and inter-assay coefficients of variation lack harmonization (Andreasson et al., 2015). These inconsistencies reduce cross-study comparability and hinder inclusion of S100A12 in multimarker panels (Ortín-Bustillo et al., 2023). To facilitate multicenter validation and clinical translation, future studies should establish standardized, traceable assays linked to reference measurement procedures, harmonized preanalytical protocols, and external quality assessment (EQA) programs to ensure analytical reliability across laboratories.

### 6.5 Summary

Although S100A12 represents a promising biomarker and therapeutic target in SA-AKI, its clinical translation is currently constrained by multiple interrelated gaps: heterogeneity in study design and endpoints (Legrand et al., 2024), limited prospective multicenter validation (Llitjos et al., 2024), species-specific barriers to translation (Hofmann Bowman et al., 2011), and the absence of standardized detection protocols (Ng et al., 2025). Addressing these limitations will require: (i) large, prospective multicenter trials with harmonized outcome definitions and prespecified sampling windows; (ii) development and cross-validation of standardized assays with preanalytical standard operating procedures (SOPs) and external quality assessment (EQA); (iii) integration of S100A12 quantification into multi-omics and multimarker frameworks to clarify biological and clinical context; and (iv) improved translational platforms—such as humanized or organoid models—to enable mechanism-informed therapeutic development. Collectively, these coordinated efforts are essential for the reliable incorporation of S100A12 into precision diagnostic and therapeutic strategies for SA-AKI.

## 7 Perspectives

Despite growing evidence implicating S100A12 in the pathogenesis and progression of SA-AKI, emerging concepts now provide a roadmap for translating this knowledge into precision diagnostics and therapeutic interventions (Legrand et al., 2024; Llitjos et al., 2024).

### 7.1 Precision medicine and enrichment trials

S100A12 is a promising molecular biomarker for patient stratification in SA-AKI. Its dynamic expression reflects neutrophil activation, inflammasome signaling, and renal tubular injury, identifying patients with pronounced inflammatory and immune-driven phenotypes (Ortín-Bustillo et al., 2023; Wu F. et al., 2025). Incorporating S100A12 into enrichment trial designs may allow selection of sub-populations most likely to benefit from immunomodulatory or anti-inflammatory therapies, thereby reducing heterogeneity in treatment responses. Endotype-guided approaches, in which patients are stratified using biomarker panels including S100A12, NGAL, and [TIMP-2]·[IGFBP7], can optimize trial efficiency and enhance the detection of clinically meaningful effects in early-stage SA-AKI and high-risk septic shock cohorts (Legrand et al., 2024; Llitjos et al., 2024).

### 7.2 Multi-omics approaches to integrate S100A12 biology

Integration of S100A12 measurements with multi-omics datasets, including transcriptomics, proteomics, metabolomics, and single-cell immunophenotyping, provides a comprehensive perspective on its role in SA-AKI pathophysiology (He et al., 2024; Zhang, 2024). Machine learning applied to these data can identify molecular signatures associated with S100A12-driven pathways, such as RAGE-mediated NF-κB and PI3K/Akt activation, NLRP3 inflammasome priming, and metabolic reprogramming of renal tubular cells. Such integrated analyses may uncover novel druggable targets, enable early detection of renal injury, and guide personalized therapeutic interventions (He et al., 2024; Zhang, 2024).

### 7.3 Potential role in guiding immunomodulatory therapies

As a DAMP and potent amplifier of inflammatory cascades, S100A12 may serve as both a biomarker and a therapeutic target (Hofmann Bowman et al., 2011; Wu F. et al., 2025). Potential strategies include direct neutralization of S100A12, blockade of its receptor RAGE, or downstream inhibition of inflammasome and NF-κB signaling. Monitoring circulating S100A12 levels could also inform the timing and dosing of immunomodulatory therapies, allowing dynamic adjustments based on the patient’s inflammatory status. Combination strategies integrating metabolic modulation with immunotherapy may further enhance tubular resilience and improve renal outcomes in SA-AKI (Hofmann Bowman et al., 2011; Wu F. et al., 2025).

### 7.4 Future directions

Future research should prioritize: (i) prospective multicenter trials incorporating S100A12 for patient stratification; (ii) development of standardized, high-sensitivity assays for longitudinal monitoring; (iii) integration of S100A12 into multi-omics frameworks to clarify mechanistic links with tubular metabolism, inflammasome activation, and apoptosis; and (iv) preclinical testing in humanized or primate models to validate therapeutic targets. Collectively, these strategies have the potential to establish S100A12 as a cornerstone of precision-guided diagnostics and immunomodulatory therapy in SA-AKI.

## 8 Conclusion

S100A12 has emerged as a mechanistically informed and clinically relevant biomarker in SA-AKI. Its rapid induction in neutrophils and monocytes, coupled with engagement of RAGE/NF-κB and NLRP3 inflammasome pathways, links systemic inflammation to tubular injury, pyroptosis and apoptosis, and metabolic dysregulation (Legrand et al., 2024; Llitjos et al., 2024; Wu F. et al., 2025). Unlike conventional markers such as serum creatinine or blood urea nitrogen (BUN), S100A12 rises early, reflecting immune activation and renal inflammatory burden, offering potential for early detection, risk stratification, and monitoring of disease progression (Ortín-Bustillo et al., 2023; Legrand et al., 2024).

Preclinical evidence supports S100A12 as a therapeutic target; neutralization, RAGE blockade, or downstream pathway modulation can mitigate tubular injury, restore metabolic homeostasis, and improve survival in experimental sepsis models (Hofmann Bowman et al., 2011; Wu F. et al., 2025). Adjunctive strategies, including immunomodulation, metabolic preprogramming, and extracorporeal removal of alarmins, may further leverage the S100A12 axis to reduce organ dysfunction (He et al., 2024; Wu F. et al., 2025).

Clinical translation remains in its early stages. Large-scale, prospective studies are required to validate predictive and prognostic utility, standardize assays, and evaluate interventions targeting the S100A12-RAGE pathway. Integration into multi-omics analyses, precision medicine frameworks, and enrichment trial designs will be critical to identify patient subphenotypes most likely to benefit (Hofmann Bowman et al., 2011; Agrawal et al., 2018; Ortín-Bustillo et al., 2023; He et al., 2024; Legrand et al., 2024; Llitjos et al., 2024; Wu F. et al., 2025).

In conclusion, S100A12 represents a mechanistic nexus connecting systemic inflammation to kidney injury in sepsis. Its dual role as an early biomarker and potential therapeutic target positions it at the forefront of precision-guided strategies for SA-AKI, providing a translationally actionable path to improve early diagnosis, risk stratification, and patient outcomes. Future research should prioritize prospective multicenter validation, assay standardization, and mechanism-driven clinical trials to accelerate S100A12-guided interventions into practice.

## Glossary

AKI: acute kidney injury
AP-1: activator protein-1
ATP: adenosine triphosphate
BUN: blood urea nitrogen
C/EBPβ: CCAAT/enhancer-binding protein beta
DAMP: damage-associated molecular pattern
ELISA: enzyme-linked immunosorbent assay
EN-RAGE: extracellular newly identified RAGE-binding protein
ERK1/2: extracellular signal-regulated kinase 1/2
ICAM-1: intercellular adhesion molecule-1
ICU: intensive care unit
IL-1β: interleukin-1 beta
IL-6: interleukin-6
IL-18: interleukin-18
KIM-1: kidney injury molecule-1
LPS: lipopolysaccharide
MAPK: mitogen-activated protein kinase
MyD88: myeloid differentiation primary response 88
NGAL: neutrophil gelatinase-associated lipocalin
NF-κB: nuclear factor kappa-light-chain-enhancer of activated B cells
NLRP3: NOD- LRR- and pyrin domain-containing protein 3
p38: p38 mitogen-activated protein kinase
PI3K: phosphatidylinositol 3-kinase
PTM(s): post-translational modification(s)
RAGE: receptor for advanced glycation end-products
ROS: reactive oxygen species; sRAGE, soluble receptor for advanced glycation end-products
SA-AKI: sepsis-associated acute kidney injury
S100A8/A9: heterodimer of S100A8 and S100A9
siRNA: small interfering RNA
SOP(s): standard operating procedure(s)
TLR4: toll-like receptor 4
TNF-α: tumor necrosis factor alpha
VCAM-1: vascular cell adhesion molecule-1
[TIMP-2]·[IGFBP7]: (tissue inhibitor of metalloproteinases-2 × insulin-like growth factor-binding protein 7).
